# Round the Hospitals

**Published:** 1916-08-26

**Authors:** 


### ROUND THE HOSPITALS.

A FEW years ago the Metropolitan Hospital Sunday Fund agreed to set apart a certain proportion of the collections for distribution amongst district nursing associations and other nursing institutions which visit patients in their own homes. We have reason to think that no money distributed by the Sunday Fund has done greater good than that given to nursing bodies. Owing to the war the number of beds available for civil patients has been materially reduced, and in some London and a few provincial hospitals the waiting lists have extended to a length which represents an amount of mental, and possibly physical, suffering to innocent people which it is painful to realise. To the honour of many hospitals the waiting lists have been reduced to almost *nil*. The ideal administration for a hospital is that which secures the adequate treatment of the greatest number of patients in the fewest number of beds in the shortest possible time. If hospital managers throughout the Metropolis would give their prompt attention to the bearings of district nursing and the relations which ought to subsist between the hospitals and all agencies supplying nurses to the homes of the sick poor, hospital beds might be made even more useful than they are at present. The best way to effect this result, experience proves, is to bring the almoner into direct touch with all in-patients who will require, or who would benefit by, free nursing attendance when they leave the hospital for their homes. It has been said that this plan means more cost, but we propose to show in a subsequent article that the sum involved is small, and that it cannot properly be regarded as an item which any smartly administered voluntary hospital can afford to omit from its accounts. To forbid this expenditure is not to save by reducing legitimate expenditure. Such a payment, on the contrary, is, in these times especially, as essential to efficient administration, in the case of a London hospital, as the cost of drugs, food, and other necessaries. The almoners, from their knowledge of district nursing work, should be able to secure for each hospital patient when at home the services of a fully-trained nurse, a service of no small importance to the poor in the Metropolis, as experience teaches.

---

Miss Haughton, matron of Guy's Hospital, is doing yeoman service on behalf of the College of Nursing. She spoke at the Leicester Royal Infirmary on the 15th instant and at the Chelsea Infirmary on the 19th. Her statements are always clear and understandable, a great point in matters involving many and technical details. Miss Gibson, of Poor-Law fame, is also doing useful service for the College. Miss Vincent and Miss Barton, the respective hostesses, are entitled to sincere acknowledgment for their services in this connection. Propaganda on behalf of the College throughout the country is essential at the moment owing to the absorbing interest taken by everybody in the war.

---

It is a great pleasure to testify to the international value of the splendid work done by English nurses in Italy under the British Red Cross. The organisation of these ambulances and the whole arrangements and equipment are, we understand, remarkably good. They have won the attention of the King of Italy and the Heir Apparent, who have specially inspected the work, and expressed the greatest pleasure and satisfaction with it and all they saw. Women have done and are doing great things in this way, and we congratulate Miss Power, the present sister-in-charge, and Miss Price, the assistant matron of the Paddington Poor-Law Infirmary, the former sister-in-charge, on the excellence of their work, which in the latter's case has conferred honour upon the Poor-Law nursing system in this country. Their work must tend materially to unite us as a nation more closely to our Italian Allies, whose prowess and patriotism long ago won the hearts of the British people.

---

The report of the Nightingale Fund for last year, just issued, states that the number of beds for soldiers now amounts to 530. The nursing arrangements for the huts were placed under the Territorial Force Nursing Service, and the governors of St. Thomas's Hospital agreed that any nurse on completion of her third year in hospital might receive her certificate should she wish to join the Territorial Force Nursing Service in the military portion of the hospital, known as No. 5 London, City of London, General Hospital. Twenty-three nurses have availed themselves of this privilege, and the unit is worked by a nursing staff numbering 107, constituted as follows: One principal matron, one matron, thirteen sisters, forty-six nurses, forty-six war probationers. The admission of ladies for short training to qualify them for work in military hospitals as war probationers at St. Thomas's Hospital resulted in applications from 1,500 candidates, of whom 167 were taken for three months' work in the civil wards. Grateful thanks are extended to the civil ward sisters for the great trouble they have taken in this training, a number of these probationers being now at work in the military huts. We congratulate Miss Lloyd Still that under her able guidance the Nightingale School has been able to retain the continuity of its system of training, lectures, and examinations for the Nightingale probationers without any disturbance due to war conditions.

---

A letter from a V.A.D. attached to the British Expeditionary Force somewhere in France appeared in the *Nursing Mirror* of the 19th instant. It reveals a weakness in the system of control over these workers which, owing to their numbers, was unavoidable at first. According to the writer's account this V.A.D., without supervision and on her own initiative, did several things which not even a trained nurse of the utmost competence would have attempted except under medical direction. We are glad to know that the authorities have this matter well in hand, and that there is good reason to believe that the steps taken will prevent any recurrence of similar vagaries on the part of an enthusiastic volunteer whose temerity was apparently only exceeded by her ignorance and absence of any feeling of responsibility. The mention of this case gives us a welcome opportunity to testify to the splendid service which the majority of V.A.D. workers have done and are doing in connection with the war. They have proved for the most part to be devoted, capable, disciplined, God-fearing women of the highest type. Their sense of duty and self-denying devotion to its discharge will go down to history as a memorable outcome of the great war which has raised the standard of womanhood and joined all classes and both sexes together in the bonds of mutual respect and confidence throughout the Empire.

**For Matrons' and Sisters' Appointments and College of Nursing Notices see p. vi.**
**For King George Hospital see p. 495**

